# The working alliance in a randomized controlled trial comparing online with face-to-face cognitive-behavioral therapy for depression

**DOI:** 10.1186/1471-244X-11-189

**Published:** 2011-12-06

**Authors:** Barbara Preschl, Andreas Maercker, Birgit Wagner

**Affiliations:** 1 Department of Psychopathology and Clinical Intervention, University of Zurich, Binzmühlestr. 14/17, 8050 Zürich, Switzerland; 2 Clinic for Psychotherapy and Psychosomatic Medicine, University Hospital Leipzig, Semmelweisstr. 10, 04103 Leipzig, Germany

## Abstract

**Background:**

Although numerous efficacy studies in recent years have found internet-based interventions for depression to be effective, there has been scant consideration of therapeutic process factors in the online setting. In face-to face therapy, the quality of the working alliance explains variance in treatment outcome. However, little is yet known about the impact of the working alliance in internet-based interventions, particularly as compared with face-to-face therapy.

**Methods:**

This study explored the working alliance between client and therapist in the middle and at the end of a cognitive-behavioral intervention for depression. The participants were randomized to an internet-based treatment group (n = 25) or face-to-face group (n = 28). Both groups received the same cognitive behavioral therapy over an 8-week timeframe. Participants completed the Beck Depression Inventory (BDI) post-treatment and the Working Alliance Inventory at mid- and post- treatment. Therapists completed the therapist version of the Working Alliance Inventory at post-treatment.

**Results:**

With the exception of therapists' ratings of the tasks subscale, which were significantly higher in the online group, the two groups' ratings of the working alliance did not differ significantly. Further, significant correlations were found between clients' ratings of the working alliance and therapy outcome at post-treatment in the online group and at both mid- and post-treatment in the face-to-face group. Correlation analysis revealed that the working alliance ratings did not significantly predict the BDI residual gain score in either group.

**Conclusions:**

Contrary to what might have been expected, the working alliance in the online group was comparable to that in the face-to-face group. However, the results showed no significant relations between the BDI residual gain score and the working alliance ratings in either group.

**Trial registration:**

ACTRN12611000563965

## Background

In the past decade, accumulating research has demonstrated that internet-based interventions can have beneficial effects on psychological health [1]. There is particular interest in the use of new communications technologies for the treatment of depression. Adult depression has a high prevalence in the general population; it is associated with significant impairments in health and functional status, as well as with high economic costs [2]. Effective and cost-efficient treatment approaches that reach large populations are therefore needed.

Internet-based interventions for depression can be delivered in different forms, from self-help treatments delivered without therapist guidance to mainly text-based interventions with high therapist involvement [3,4]. However, research indicates that the treatment outcomes of internet-based interventions are related to amount of therapist involvement. In their meta-analysis of internet-based interventions for depression, Andersson and Cuijpers [5] found a strong influence of therapist support on treatment outcome. Computerized interventions with therapist support showed a mean between-group effect size of d = .61, which is comparable with face-to-face treatment for depression, whereas interventions with little or no therapist contact had a significantly smaller treatment effect size of d = 0.25. This pattern of results replicates the findings of a previously published meta-analysis [6]. Moreover, studies on entirely self-guided programs have shown not only reduced treatment effects, but also substantial attrition rates of up to 41% [7-11]. Analyses have also revealed a significant correlation between the amount of therapist time in minutes per participant and the between-group effect sizes of internet-based interventions [12]. Based on the findings of their Swedish studies, Andersson and colleagues have suggested that it can be sufficient for the therapist to spend about 100 minutes per patient over a 10-week program giving comments on patients' homework and providing feedback [13]. The latest studies indicate that increasing therapist contact time beyond a certain threshold may not facilitate further treatment gains [14]. In his review, Titov [15] concluded that highly standardized internet-based interventions with low-intensity therapist support can achieve excellent clinical outcomes. Overall, these studies on internet-based interventions for depression thus suggest that a minimum of human therapeutic contact is needed to reduce attrition rates and to alleviate symptoms of depression.

Despite the growing interest in the influence of therapist support (e.g., therapist time spent per patient) in internet-based interventions, there has been little research on therapeutic process factors and predictors of treatment outcome in online settings. It therefore remains unclear whether the factors and therapeutic processes that are responsible for symptom reduction in face-to-face therapy operate in the same way in online therapeutic settings. We expect more factors to be involved than the mere amount of time that the therapist spends giving feedback to patients.

### Therapeutic alliance

One of the therapeutic process factors associated with treatment outcome is the working alliance between therapist and patient. Numerous empirical studies have demonstrated the importance of the working alliance--that is, the relationship or collaboration between therapist and patient--for therapeutic outcomes in conventional treatment settings [16]. It has also been noted that clients' assessments of the therapeutic alliance are more predictive than are therapists' or observers' ratings. Krupnick and colleagues [17] demonstrated that the therapeutic alliance significantly influenced symptoms of depression as outcome measures. They found significant predictive effects for patient ratings, but not for therapist ratings. In view of these findings, the therapeutic alliance has traditionally been seen as a key element adding to the treatment success of face-to-face psychotherapy [16]. Against this background, the fact that internet-based interventions involve less therapeutic contact--not only in terms of time, but also through their restriction to purely text-based and computer-mediated communication--may be a cause for concern. However, there has to date been little empirical research on the impact of the working alliance in online settings as compared with face-to face therapeutic settings.

Cook and colleagues [18] were among the first to evaluate the online working alliance. They compared results from an online sample (*N = *15) with normative data from a representative sample in face-to-face therapy (*N = *25). The online group showed higher means on the composite score and the goals subscale of the Working Alliance Inventory [19]. The goals subscale reflects the agreement between therapist and client on what is to be achieved in the therapy. However, these preliminary results should be interpreted carefully: the sample size was small and patients were not randomly allocated to the conditions. In the same vein, Reynolds and colleagues [20] reported preliminary results (*N *= 16 therapists, *N *= 17 clients) on the therapeutic alliance as assessed by the Agnew Relationship Measure [21] in an online setting, which they compared with existing data from a face-to-face group. The clients in the online study presented with depression, stress, anxiety, or childhood abuse. Like Cook and Doyle [18], the authors reported similar therapeutic alliance ratings for both conditions, with the online groups showing higher means on the confidence subscale. In a randomized controlled study, Knaevelsrud and Maercker [22] compared the therapeutic alliance in a total of 96 PTSD patients assigned at random to an internet-based treatment or a waiting list control group. The treatment involved 10 writing assignments, on which therapists gave detailed feedback. The authors reported relatively low drop-out rates (16%) and relatively high scores for the therapeutic alliance (Working Alliance Inventory, patient ratings: M = 6.3, therapist ratings: M = 5.8). These results were again comparable with face-to-face therapy, indicating that a strong therapeutic relationship could be established even in an online setting with no direct personal contact. Further, the composite scores of both the therapists' and the clients' ratings of the therapeutic alliance late in treatment were moderately but not significantly correlated with treatment outcome [23].

Beside these studies of internet-supported therapeutic interventions with therapist support based on computer-mediated communication without the use of a specific self-help program, Klein and colleagues [24] and Kiropoulos and colleagues [25] have reported positive results on the therapeutic alliance in therapist-assisted internet programs. In a randomized controlled trial, Kiropoulos and colleagues compared a 12-week internet-based cognitive behavioral therapy (CBT) for panic disorder and agoraphobia provided via the online program *Panic Online *with face-to-face CBT (*N = *86). The program combines standardized instructions and information with e-mail contact with a therapist. Patients in the internet-based group had significantly less therapist contact than those in the face-to-face group. Nevertheless, both groups rated the intervention as similarly satisfying (Treatment Satisfaction Questionnaire-Modified, TSQ; [26]) and credible (Treatment Credibility Scale, TCS-M; [27]). However, participants in the face-to-face group enjoyed communication with their therapist more than did those in the internet-based group, and their therapists reported higher compliance to treatment (Therapist Alliance Questionnaire, TAQ; modified version of the Helping Alliance Questionnaire, HAQ; [28]). In an open trial, Klein and colleagues investigated a therapist-assisted internet CBT for PTSD provided via the interactive CBT program *PTSD Online*. These authors reported 194.5 min of therapist time spent across the 10-week intervention. Nevertheless, the participants (*N = *22) gave high therapeutic alliance ratings (87.5%) on the Therapeutic Alliance Questionnaire, TAQ.

Based on these findings, we conducted a randomized controlled study investigating the therapeutic alliance in online (computer-mediated communication without the use of a specific self-help program) and face-to-face CBT treatment settings for depression. To our knowledge, this is the first randomized controlled trial for depression to compare the therapeutic alliance between patient and therapist in the two settings in an experimental design. To maximize comparability, all patients received the same treatment manual over the same timeframe. The treatment manual was based on a German CBT treatment manual for depression [29] with an added life-review intervention module [30]. The first objective of this study was to examine whether the therapeutic alliance was comparable in the online group and the face-to-face group. Second, we investigated whether the therapeutic alliance predicted depression as outcome in the online and/or face-to-face condition. Third, we examined the therapeutic alliance from the therapists' perspective as a predictor of treatment outcome in both conditions.

## Method

### Study design

A randomized controlled trial comparing an internet-based with a face-to face CBT intervention for depression was conducted at the University of Zurich [31]. Both treatment groups received the same cognitive behavioral therapy over an 8-week timeframe, at the end of which participants completed the Beck Depression Inventory and the Working Alliance Inventory. Assessments were conducted at baseline and post-treatment.

### Participants

Participants were recruited between November 2008 and February 2010. The institutional review board at the University of Zurich approved the study. Patients were recruited through advertisements in newspapers, the depression website of the university, local internet news forums, and depression self-help groups, advertisements in supermarkets and pharmacies, and local press releases. Inclusion criteria were a score of at least 12 on the Beck Depression Inventory (BDI) [32] and age 18 years or older. Demographic characteristics of the sample are presented in Table 1.

**Table 1:** Sample characteristics.

Characteristics	Total sample (*n *= 53)	Online group (*n *= 25)	Face-to-face group (*n *= 28)	Comparison (df)
Age, M (SD) (in years)	36.7 (10.9)	34.9 (9.5)	38.3 (11.9)	F(.91) = .25, ns
Gender (female)	36 (67.9%)	21 (84%)	15 (53%)	*χ^2^*(1), *p *.01
Marital status				*χ^2^*(3) = .58, ns
Single	31 (58.5%)	15 (60%)	16 (57%)	
Married/cohabiting	9 (17%)	4 (16%)	5 (18%)	
Divorced	6 (11.3%)	4 (16%)	2 (7%)	
Widowed	7 (13.2%)	2 (8%)	5 (18%)	
Educational level				*χ^2^*(2) = .21, ns
Vocational-track sec. school	11 (20.8%)	3 (12%)	8 (28%)	
Intermediate-/academic- track sec. school	20 (37.7%)	12 (48%)	8 (28%)	
University degree	22 (41.5%)	10 (40%)	12 (43%)	
Employment status				
Full-time work	36 (97.9%)	18 (72%)	18 (64.3%)	
Unemployed	11 (20.8%)	5 (20%)	6 (21.4%)	
Sick leave	4 (7.5%)	1 (4%)	3 (10.7%)	
Retired	1 (1.9%)	1 (3.6%)		
No antidepressants	43 (81.1%)	23 (92%)	20 (71%)	*χ^2^*(1) = .14, ns
Previous face-to-face psychotherapy	28 (52.8%)	13 (52%)	15 (54%)	*χ^2^*(1) = .54, ns
Visits to medical doctors in last 12 months, M (SD)	4.5 (5.3)	5.2 (5.5)	3.78 (4.9)	F(.002) = .30, ns
Where did you hear about the study?				
Internet	30 (56.6%)	13 (52%)	17 (60.7%)	
Newspaper	12 (22.6%)	5 (20%)	7 (25%)	
Radio	2 (3.8%)	2 (8%)		
Family/friends	1 (1.9%)	1 (4%)		
Self-help group	3 (5.7%)	3 (12%)		
Bulletin	4 (7.5%)	1 (4%)	3 (10.7%)	
Caregiver (psychologist, primary care physician)	1 (1.9%)		1 (3.6%)	

The average BDI baseline score was M = 22.5 (S.D = 6) for the online group and M = 23.6 (SD = 7.9) for the face-to-face group. The BDI baseline scores of the two groups did not differ significantly, *t*(50) = -0.567; *p *> .05. Information on post-treatment BDI scores and associated test statistics are reported elsewhere [31]. Preliminary results for the primary outcome (depression) revealed no differences between the online and the face-to-face condition.

### Procedure

A web page was created for the study, presenting general information about CBT and its effects in treating depression, and giving an outline of the study. Participants indicated their interest in the study by contacting the intake coordinator via the e-mail address indicated on the website (for further information, see [31]). The intake coordinator sent a reply e-mail with a patient information sheet and the inclusion and exclusion criteria. Participants who indicated that they met and were comfortable with the requirements entered an online screening procedure, data from which were later used as pretest measures. After confidentiality issues had been addressed, eligible applicants returned a signed informed consent form--which informed them about potential risks and benefits of study participation--by fax or post. The treatment commenced 3 to 4 days after the patients had returned their informed consent form. The intake coordinator told participants that they could withdraw from the study at any time. Further, participants received 24-hour contact numbers for emergency situations or crises. They were also encouraged to call or e-mail the therapist or intake coordinator at any time during their participation in the study in case of distress or crisis. Participants were randomly assigned to one of the two conditions as they were included in the study. Applicants excluded from the study were informed about other available forms of treatment.

**Figure 1:**
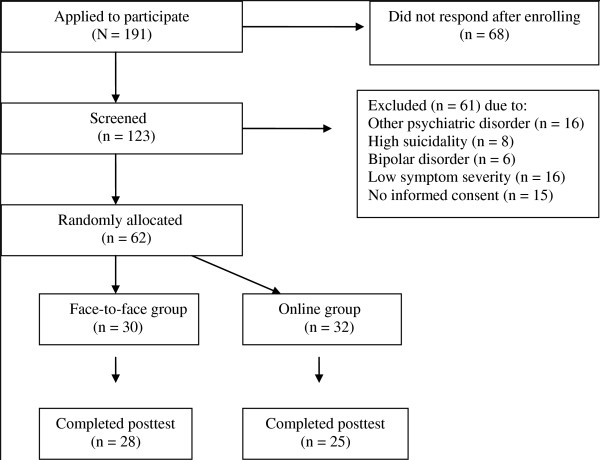
**Flowchart of participant progress**.

As shown in Figure 1, a total of 191 respondents applied for the treatment. The 62 applicants included in the study were randomized by a true random-number service (http://www.random.org), with 32 participants being randomly allocated to the online group and 30 to the face-to-face treatment group. Randomization was performed by the study coordinator and was not stratified by any participant characteristics. Seven (22%) participants in the online group and two (7%) participants in the face-to-face group failed to finish the treatment. The main reasons given for discontinuing the treatment were lack of time, sufficient improvement, and lack of motivation. Participants who dropped out of treatment were not considered in the analyses.

### Measures

All measures were self-reports administered via an online diagnostic assessment. Fidy [33] found no significant differences between paper-and-pencil and online administration of the German versions of the BDI and the Beck Suicide Ideation Scale (BSIS), which were also used in the present study. Outcome measures were administered at baseline and post-treatment. The working alliance (patients' ratings) was also assessed at mid-treatment after 4 weeks.

### Outcome measures

#### Depression

Depression was assessed with the German version [34] of the Beck Depression Inventory-II (BDI; [32]), which comprises 21 multiple-choice items assessing specific symptoms of depression. The BDI has shown high reliability across diverse populations. The internal consistency in the current sample was α = .91.

#### Working alliance

The quality of the working alliance was assessed by the German version [23] of the Working Alliance Inventory (WAI [35]). Respondents were asked to rate each statement on a 7-point Likert scale ranging from 1 (*never*) to 7 (*always*). In this study, both the client and the therapist version of the 12-item WAI-S [36] were administered at post-treatment. The WAI covers three aspects of the working alliance: bond (degree of mutual trust, acceptance, and confidence between client and therapist; client: α = .84; therapist: α = .84), tasks (agreement on therapeutic tasks; client: α = .88; therapist: α = .77), and goals (agreement on therapeutic goals; client: α = .87; therapist: α = .84). The internal consistencies for the composite scores in our sample were high (client: α = .94, therapist: α = .93).

### Exclusion criteria

Applicants were excluded if they met any of the following criteria: currently receiving treatment elsewhere, substance abuse or dependence, on antidepressant medication for less than 4 weeks, age below 18 years, not fluent in German. Further exclusion criteria were high risk of suicide, psychotic symptoms, post-traumatic stress disorder, anxiety, phobia, bipolar disorder, and low depression symptom severity.

#### Depression

Symptom severity was assessed by the German version of the Beck Depression Inventory [32]. Patients were excluded if their BDI score was below 12.

#### Suicide ideation

Suicide ideation was assessed with the Beck Suicide Ideation Scale [37], a 21-item inventory developed to measure the intensity and chronicity of suicide ideation in adults. The first 5 items make up a brief subscale measuring the presence of suicidal thoughts, either recently (in the last 6 months) or ever in one's life.

#### Risk of psychosis

Risk of psychosis was measured using the Dutch Screening Device for Psychotic Disorder [38], a seven-item inventory that is a good predictor of psychotic episodes. Because no data are yet available from a German norm group, the Dutch norm data were used.

#### Anxiety

Anxiety was assessed using the Anxiety subscale of the German version of the Symptom Checklist by Derogatis [39]. This 10-item subscale covers various symptoms of anxiety, including cognitive and somatic correlates of anxiety.

#### Phobia

The German version of the Symptom Checklist by Derogatis [39] was also used to measure phobia. The Phobia subscale contains seven items assessing severity of phobic symptoms.

#### Post-traumatic stress

The Post-traumatic Stress Scale 10 [40], a short screening instrument tapping DSM-III symptoms of post-traumatic stress disorder including symptoms of hyperarousal, was used to measure symptoms of post-traumatic stress.

### Therapists

Six female psychologists and psychotherapists participated in this study. All psychologists were trained in psychotherapy and CBT for depression specifically for this study. The therapists were given special training in therapeutic writing for the online treatment and received regular supervision (face-to-face and online), with therapists in both groups receiving the same amount of supervision. All but one of the therapists were involved in both treatment conditions. Therapists were not randomly allocated to patients.

### Treatment

Both treatment conditions were of equal length (8 weeks) and followed an evidence-based short-term CBT treatment manual for depression [29]. This German manual is based on the cognitive theory of depression by Beck and colleagues [34]. The program involved the following modules: introduction, behavioral analysis, planning of activities, daily structure, cognitive restructuring, promotion of social competence, and relapse prevention. A life-review module was added to the standard CBT treatment manual [31]. The aims of life review are to revisit and reattribute past experiences and to activate positive memories and individual resources in order to achieve a balance between positive and negative memories. In the present context, this method was essentially used to activate individual resources (e.g., to identify coping strategies that had helped participants to cope with unresolved past experiences or depressive episodes).

Patients in both groups were given the same psychoeducation and received the treatment modules in the same chronological order. Psychoeducation played an important role in the therapeutic approach. At the beginning of each new treatment module, the patient was informed about the meaning and background of each treatment technique, the significance of the homework set, and the meaning of certain symptoms or reactions.

Patients in the face-to-face condition attended one-hour weekly treatment sessions for 8 weeks with their allocated psychologist in the Department of Psychopathology and Clinical Intervention at the University of Zurich. They were also given weekly homework assignments (e.g., daily structure diaries, negative thoughts log).

For the online condition, the CBT treatment manual for depression [29] was adapted for use as an internet-based intervention, based on the principles applied in a number of previous studies [3,41-43]. To this end, a highly structured treatment manual was developed. The treatment consisted of structured writing and homework assignments (e.g., behavioral analysis of depressive symptoms, activity diaries, cognitive restructuring worksheets) based on the CBT approach and on the written disclosure procedure developed by Pennebaker and colleagues [3,44]. Each writing assignment lasted 45 minutes and took place at regular, scheduled times. Within one working day, the therapist provided individual written feedback along with instructions on the next writing assignment. Model responses for the therapists were available, but they also had the option to provide their own commentary or supportive feedback on their patients' texts. Patients were given two writing assignments in each week of the 8-week treatment period. The therapist time involved in responding to texts ranged from 20 to 50 minutes per text, depending on the therapist's experience with internet-based therapies.

### Data analysis

SPSS 17.0 for Windows was used for all analyses. In preliminary analyses, we compared the online and face-to-face group at baseline using *t *and chi-square tests. *T *tests were then used to compare the therapeutic alliance in the two intervention groups. In addition, bivariate and partial correlations (Pearson) were calculated to examine the relationship between the working alliance and therapy outcome.

Treatment outcome was assessed as (a) the BDI score at post-treatment (BDI-post) and (b) the BDI residual gain score (the difference between the z-transformed BDI scores at post-treatment and baseline multiplied by the correlation between the two scores [45]). The therapeutic alliance was assessed in terms of the composite score on the WAI and the scores on the three subscales (bond, tasks, goals) of the clients' (WAI-C) and the therapists' (WAI-T) ratings.

To quantify the magnitude of differences between the two groups (online versus face-to-face), we used Cohen's *d *as a measure of effect size. Cohen [46] distinguished between small (*d *= .20), medium (*d *= .50) and large (*d *= .80) effect sizes.

Since we did at no time obtain data concerning therapeutic alliance from drop outs we could not conduct intention-to-treat analysis.

## Results

### Quality of the working alliance in the treatment groups

Table 2 shows the means, standard deviations, *p *values (*t *tests), and effect sizes for the quality of the working alliance in the online and the face-to-face group. Patients and therapists were asked to evaluate the quality of the working alliance at post-treatment; patients additionally completed the Working Alliance Inventory at mid-treatment after 4 weeks. Ratings were given on a scale from 1 to 7, with high values indicating a strong therapeutic alliance. As shown in Table 2, in the online condition, the clients' post-treatment ratings (WAI-C) tended to be slightly higher than the therapists' post-treatment ratings (WAI-T). Further, the subscale and composite scores of both the WAI-C and the WAI-T were all slightly higher in the online condition than in the face-to-face condition. However, with the exception of the WAI-T tasks score, which was significantly higher in the online condition (*p *< 0.05), the differences between the online and the face-to-face groups were not significant.

**Table 2:** Working alliance in the two intervention groups: means, standard deviations, t test comparisons, and effect sizes.

	Sample means								
	Online (*N *= 25)			Face-to-Face (*N *= 28)					
Scales	*M*	*SD*	*n*	*M*	*SD*	*n*	*Test statistic*	*p*	*d*
WAI-C mid-treatment									
Tasks	5.82	.80	25	5.48	1.05	28	1.30^4^	.19	0.36
Bond	5.68	.99	25	5.39	1.01	28	1.04^5^	.30	0.29
Goals	5.95	.81	25	5.81	1.01	28	.54^5^	.59	0.15
Composite	5.82	.78	25	5.56	.91	28	1.08^5^	.28	0.31
WAI-C post-treatment									
Tasks	6.17	.80	25	5.66	1.18	27	1.81^1^	.07	0.51
Bond	5.91	.97	25	5.67	.95	28	.91^4^	.36	0.25
Goals	6.22	.79	25	5.98	1.01	28	.94^5^	.35	0.26
Composite	6.10	.77	25	5.76	.98	27	1.39^3^	.16	0.39
WAI-T post-treatment									
Tasks	6.16	.60	25	5.66	.89	28	2.36^2^	.02*	0.66
Bond	5.86	.90	25	5.79	1.14	28	.26^5^	.79	0.07
Goals	6.11	.71	25	5.98	1.00	28	.53^3^	.59	0.15
Composite	6.04	.67	25	5.80	.96	28	1.01^3^	.31	0.29

WAI-C = Working Alliance Inventory - Clients' ratings; WAI-T = Working Alliance Inventory - Therapists' ratings; ^1 ^df = 46, ^2 ^df = 47, ^3 ^df = 48, ^4 ^df = 49, ^5 ^df = 50.

* *p *< .05.

### Working alliance and therapy outcome

Table 3 shows the correlations of the WAI scores at mid- and post-treatment with the BDI score at post-treatment and the BDI residual gain score. Significant correlations were found between therapy outcome and clients' ratings of the working alliance in the online group (tasks subscale) at post-treatment and in the face-to-face group at mid- (tasks subscale and composite score) and post-treatment (tasks, goals, and composite scores). The BDI baseline score was included in the analysis as a control variable. Further, analysis of the relations between the BDI residual gain score and the WAI scores revealed that the working alliance ratings did not significantly predict the BDI residual gain score in either group at mid- or post-treatment.

**Table 3:** Correlations of the WAI scores with the BDI score at post-treatment and the BDI residual gain score in the online and face-to-face groups.

Variable	BDI-post (covariate: BDI-pre)		BDI residual gain score	
	Online	Face-to-face	Online	Face-to-face
WAI-C mid-treatment				
Tasks	-.16	-.52**	-.15	-.35
Bond	-.04	-.21	.12	-.08
Goals	-.09	-.32	-.16	-.22
Composite	-.10	-.40*	-.06	-.24
WAI-C post-treatment				
Tasks	-.47*	-.46*-.	-.33	-.33
Bond	-.15	-.27	.08	-.22
Goals	-.36	-.43*	-.33	-.33
Composite	-.35	-.42*	-.20	-.32
WAI-T post-treatment				
Tasks	-.25	-.29	-.12	-.16
Bond	-.07	-.20	.16	-.15
Goals	-.24	-.22	-.11	-.13
Composite	-.20	-.24	-.01	-.14

WAI-C = Working Alliance Inventory - Clients' ratings; WAI-T = Working Alliance Inventory - Therapists' ratings; BDI = Beck Depression Inventory.

* *p *< .05. ** *p *< .01.

## Discussion

The aim of this study was to investigate the quality of the therapeutic alliance between patient and therapist in an online and face-to-face CBT for depression. To our knowledge, this was the first randomized controlled trial in this context. First, we examined whether the therapeutic alliance was comparable in both groups. Our results showed that the online and the face-to-face group differed significantly in only one subscale: therapists' ratings of the tasks subscale were significantly higher in the online group. This finding is in line with previous studies reporting that a strong working alliance, comparable to that formed in face-to-face settings, can also be established in online settings. The WAI mean scores in our study ranged from 5.39 to 6.22 (of a maximum of 7). These findings are comparable to data presented by Knaevelsrud and Maercker [22], who reported mean scores ranging from 5.6 to 6.4 in Table 3 of their article. Furthermore, authors using other scales or other versions of the WAI have also provided evidence for a comparably strong working alliance in online settings as in face-to-face therapy. Cook and Doyle [18], for example, reported results for an online sample to be comparable with normative data from a representative sample in face-to-face therapy. Most of the participants in their sample presented with relationship issues, depression, anxiety, or grief. However, because of the small sample size and the non-randomized allocation of patients, these preliminary results should be interpreted with caution. In the same vein, Reynolds and colleagues [20] reported ratings of the therapeutic alliance in an online setting to be similar to existing data from a face-to-face group. The participants in their study presented with depression, stress, anxiety, or childhood abuse. We were able to replicate the findings from both of these studies in a randomized controlled setting with a sample of depressive adults. The higher therapist ratings of the tasks subscale in the online group in our study may be attributable to the clear presentation and structuring of the tasks in the online mode, and to the opportunity to focus carefully on elaborated tasks. This fact may have positively influenced the agreement between clients and therapists on the therapeutic tasks.

Further, the drop-out rate in our study was relatively low. Seven (22%) participants in the online group and two (7%) participants in the face-to-face group discontinued the treatment. In general, drop-out rates in internet-based interventions are known to be problematic [5]. However, the drop-out rates reported for studies involving internet-based interventions for depression over the last five years differ widely. For instance, Titov and colleagues [47] reported that 11% of participants in a clinician-assisted internet-delivered CBT for depression did not complete post treatment questionnaires. In contrast, Spek and colleagues [48] reported a drop-out rate of 66% for the intervention group of an internet-based CBT intervention study for subthreshold depression (individuals who did not complete post-test, did not start the intervention, or withdrew). In our sample, the attrition rates in the online group (22%) versus the face-to face group (7%) differed significantly, *χ^2^*(1) = 4.737, *p *.05. This may indicate that the more anonymous online therapeutic relationship is less stable than the face-to-face relationship. It is easier for patients in online treatment settings to stop therapeutic communication by simply "disappearing." A study of online romantic relationships revealed that avoidance behavior and discontinuity are more likely in online relationships than in face-to face relationships [49].

Furthermore, we were interested in whether the therapeutic alliance predicted depression as outcome in the online or the face-to-face group. In both groups, only the clients' ratings of the working alliance were associated with depression at post-treatment (specifically, the composite score and tasks subscale in the face-to-face group at mid-treatment and, at post-treatment, the tasks subscale in the online group and the composite score and the tasks and goals subscales in the face-to-face group). It is worth noting that the correlations reported here are statistically significant, but only moderately high, ranging from *r *= -.42 to *r *= .52. These results are in line with findings on face-to-face psychotherapy. In a review article, Martin and colleagues [50] reported a moderate but consistent relationship between the therapeutic alliance and outcomes of face-to-face psychotherapy. However, in the online group, only the working alliance at post-treatment was significantly associated with depression at post-treatment. This result replicates the findings of Knaevelsrud and Maercker [23], who found no significant relationship between the working alliance at mid-treatment and PTSD change scores. Further, our data showed no significant relations between the BDI residual gain score and the working alliance in either group at mid- or post-treatment. Knaevelsrud and Maercker [22] discussed the importance of investigating the working alliance at several stages of the therapeutic process to elucidate the relationship between working alliance and outcome. The authors suggested that the working alliance might be more an "additional indirect measure of outcome" than a predictor of treatment outcome.

The limitations of our study include the assessment of the working alliance and depression. As participants were first contacted online and later allocated at random to the online or the face-to-face group, all measures were administered as self-rated questionnaires in an online setting. Although this procedure has proven valid and reliable in various previous studies [18,20,22-25], a structured clinical interview would have allowed a better quality of diagnosis of depression and the therapeutic relationship.

A further limitation is that we are unable to present follow-up data at the present time. Collection of follow-up data (after 3, 6 and 12 months) is still ongoing. Therefore, it remains an open question whether the working alliance at post-treatment predicts outcomes at follow-up.

Furthermore, the sample used in this study was small, relatively well educated and more than half of the participants already had experience of psychotherapy. Future studies should enroll larger and more heterogeneous samples. Another limitation of the study is the generalizability of our results. Due to our strict exclusion criteria regarding co-morbidity, suicide ideation, and psychosis, a number of applicants were excluded from the study. Our findings may therefore not be comparable with more naturalistic designs. Further research is needed to focus specifically on patients with co-morbidities.

## Conclusions

In conclusion, an internet-based intervention has the potential to facilitate a working alliance that is comparable to that formed in face-to-face settings, though not as influential with respect to symptom reduction. This is the first randomized controlled trial to evaluate the therapeutic alliance between patient and therapist in online and face-to-face treatment settings for depression in an experimental design. Our study contributes to a better understanding of the working alliance in internet-supported therapeutic interventions, replicating previous findings [18,20,22-25] showing that a strong working alliance can be established in an online setting, comparable to that established in face-to-face settings.

## Competing interests

The authors declare that they have no competing interests.

## Authors' contributions

BW and AM planned and initiated the study. BW and BP carried out analysis and interpretation of data, and drafted the manuscript. All authors read and approved the final manuscript.

## Pre-publication history

The pre-publication history for this paper can be accessed here:

http://www.biomedcentral.com/1471-244X/11/189/prepub
